# MASCC/ISOO Clinical Practice Statement: The risk of secondary oral cancer following hematopoietic cell transplantation

**DOI:** 10.1007/s00520-024-08685-y

**Published:** 2024-07-25

**Authors:** Judith E. Raber-Durlacher, Nathaniel S. Treister, Yehuda Zadik, David R. Dean, Wanessa Miranda-Silva, Eduardo R. Fregnani, Joel B. Epstein, Sharon Elad

**Affiliations:** 1 Department of Oral Medicine, Academic Center for Dentistry Amsterdam (ACTA), Department of Oral and Maxillofacial Surgery, Amsterdam UMC, University of Amsterdam and VU University, University of Amsterdam, Gustav Mahlerlaan 3004, 1081 LA Amsterdam, The Netherlands; 2 Division of Oral Medicine and Dentistry Brigham and Women’s Hospital and Department of Oral Medicine, Infection and Immunity, Harvard School of Dental Medicine, Boston, MA USA; 3 Department of Oral Medicine and Saligman Clinics, Faculty of Dental Medicine, The Hebrew University of Jerusalem, and Hadassah Medical Center, Jerusalem, Israel; 4 Department of Oral Medicine, University of Washington School of Dentistry and Fred Hutchinson Cancer Center, Seattle, WA USA; 5 Centro de Oncologia Molecular, Instituto de Ensino e Pesquisa, Hospital Sírio-Libanês, São Paulo, SP Brazil; 6 Dental Oncology Service, City of Hope Comprehensive Cancer Center, Duarte, CA and Cedars-Sinai Health System, Los Angeles, CA USA; 7 Oral Medicine, Eastman Institute for Oral Health, University of Rochester Medical Center, Rochester, NY USA

**Keywords:** Hematopoietic cell transplantation, Graft versus host disease, Oral care, Risk factors, Secondary oral cancer

## Abstract

**Purpose:**

A MASCC/ISOO Clinical Practice Statement (CPS) is aimed at generating a concise tool for clinicians that concentrates practical information needed for the management of oral complications of cancer patients. This CPS is focused on the risk of secondary oral cancer following hematopoietic cell transplantation (HCT).

**Methods:**

This CPS was developed based on critical evaluation of the literature followed by a structured discussion of a group of leading experts, members of the Oral Care Study Group of MASCC/ISOO. The information is presented in the form of succinct bullets to generate a short manual about the best standard of care.

**Results:**

Studies described a 7–16-fold higher risk of secondary oral cancer (mainly squamous cell carcinoma) in allogeneic HCT (alloHCT) recipients, particularly in those who developed chronic graft versus host disease (cGVHD). Risk increases over time and is influenced by several risk factors. In autologous HCT, oral cancer risk seemed only slightly elevated.

**Conclusion:**

Clinicians should be aware of the higher oral cancer risk in alloHCT survivors, and emphasize the importance of lifelong oral cancer surveillance (at least every 6–12 months) and avoiding cancer promoting lifestyle factors in an empathic way, particularly of those with (a history of) cGVHD. Post-HCT for Fanconi anemia or dyskeratosis congenita, education and rigorous follow-up is even more crucial. In case of suspected oral lesions in the presence of oral mucosal cGVHD, a GVHD intervention may facilitate diagnosis. Suspected lesions should be biopsied. More research is needed on the role of HPV in oral cancer post-HCT.

**Supplementary Information:**

The online version contains supplementary material available at 10.1007/s00520-024-08685-y.

## Introduction

The number of hematopoietic cell transplantations (HCT) performed annually has increased over the last decades with improved survival [1, 2]. HCT has some specific late complications including secondary cancers, defined as an independently developing malignancy in an individual previously diagnosed with a different type of malignancy [3]. Among the secondary cancers, oral squamous cell carcinoma (SCC) is the predominant type of secondary oral cancer after HCT, although non-SCC oral malignancies such as salivary gland cancer have also been reported [4, 5]. Furthermore, oral SCC may be more aggressive than in non-transplant patients and tumors may be multifocal [6].

Large-scale studies described a higher risk of secondary oral cancer in allogeneic HCT (alloHCT) recipients, which is estimated to be approximately 7- to 16-fold of the expected risk in the general population [4, 5, 7, 8]. The risk is possibly higher in patients with chronic graft versus host disease (cGVHD) or history of cGVHD [9]. The combination of extended tissue inflammation together with prolonged immunosuppression associated with severe cGVHD and its treatment likely puts patients at risk for epithelial dysplasia and malignant transformation [10, 11]. The risk of developing oral cancer continues to rise over time [8]. In addition to cGVHD and time since transplant, other risk factors for oral SCC post-alloHCT have been reported. These include previous treatment with azathioprine, underlying diagnosis of Fanconi anemia (FA) or dyskeratosis congenita (DKC), pre-HCT cancer treatment, conditioning regimen, stem cell source, young as well as advanced age at HCT, male sex, and genetic predisposition [4, 10, 12–14]. The risk of oral cancer following reduced intensity and non-myeloablative conditioning regimen seems at least as high as in myeloablative protocols, but more long-term follow up studies are necessary to determine this risk [15]. In autologous HCT recipients, oral cancer risk seems only slightly elevated compared to the general population [16].

Given the higher risk of oral cancer, a working group of the Oral Care Study Group (OCSG) of the Multinational Association of Supportive Care in Cancer (MASCC) and the International Society of Oral Oncology (ISOO) composed an expert-opinion Clinical Practice Statement (CPS) that will provide a framework for evaluating and counseling patients, thereby leading to early detection of secondary oral cancer. In the absence of formal MASCC/ISOO guidelines on this topic, this CPS presents the Society’s view of the best standard of care.

## Objectives

To raise awareness of the higher risk for oral cancer in patients post-HCT, to promote early detection of oral cancer in HCT recipients, and to prompt a constructive conversation with the patients at high risk.

## Methods

A critical evaluation of the literature was conducted by a group of leading experts. PubMed was searched on citations pertinent to secondary oral cancer post-HCT up to January 1, 2023. The literature search did not include post-transplant lymphoproliferative disorder or manifestations of the underlying hematologic malignancy such as granulocytic sarcoma. During the development of the manuscript, point questions that deemed a closer look were generated, and a literature search was done to ensure accuracy of information. The CPS was discussed in a multi-step structured manner by an OCSG working group composed of experts on the topic of oral complications post-HCT, and then reviewed by two independent boards: ISOO Advisory Board and the MASCC Guidelines Committee. The Statement follows the MASCC/ISOO Guidelines Policy.

## Clinical relevance and practical considerations

- Clinicians should be aware of the higher risk for oral second malignancies in alloHCT survivors, and advocate for routine surveillance with an oral medicine specialist, an oral and maxillofacial surgeon or another health care professional with expertise in managing oral cancer patients. Special attention should be given to patients with cGVHD or history of cGVHD, particularly those with oral involvement.
- Survivors should be screened for oral cancer every 6–12 months lifelong, or more frequently if the health care provider or patient notices a change in the oral mucosa.
- Patients should be educated about the risk of oral cancer in order to increase compliance for lifelong cancer surveillance. The clinician should deliver this information in a non-stressful manner that will engage the patient in the follow-up plan. An empathic professional conversation is key for obtaining the patient’s collaboration.
- Patients should be informed about the importance of reducing/avoiding risk factors, including oral cancer–promoting lifestyle factors (e.g., smoking, betel nut use, alcohol abuse, sun exposure of the lips).
- In patients with suspected oral lesions in the presence of oral mucosal cGVHD, an intervention for oral cGVHD may be considered to differentiate the chronic disease from oral cancer [17].
- Biopsy and pathological examination should be performed in cases of suspicion for malignancy.
- The contribution of human papillomavirus (HPV) to risk of oral cancer post-HCT is not fully elucidated. Several studies reported of an association between HPV and oral cancer post-HCT, or evidence for higher risk for HPV-related cancer post-HCT [18, 19]. The implication of this will be revealed as more studies on the prognostic value of HPV in oral cancer post-HCT will be performed.
- Patients with FA and DKC have an increased risk of  oral cancer. The need for oral cancer surveillance plan and discouragement of smoking and alcohol use in FA or DKC patients who underwent HCT is even more crucial. Therefore, increased frequency of follow-up should be considered in this group of patients.

### Supplementary Information

Below is the link to the electronic supplementary material.

Supplementary file1 (PDF 106 KB)

## Declarations

### Disclaimer

The MASCC/ISOO OCSG Statements have been developed to facilitate the expert-opinion based management of oral complications of cancer and cancer therapy where high-quality evidence is lacking. Clinicians should use their judgment when making treatment decisions for individual patients. The Statement authors and the MASCC/ISOO do not guarantee or take responsibility for clinical outcomes in individual patients.
