# Supplementary material for: MASCC/ISOO Clinical Practice Statement: The risk of secondary oral cancer following hematopoietic cell transplantation
Source: Support Care Cancer. 2024 Jul 25;32(8):545. doi: 10.1007/s00520-024-08685-y (PMC11269467; doi:10.1007/s00520-024-08685-y)
Supplement: Supplementary file 1 — Supplementary file1 (PDF 106 KB) [file 520_2024_8685_MOESM1_ESM.pdf]

MASCC/ISOO Clinical Practice Statement:

The risk of secondary oral cancer following hematopoietic cell transplantation

Suggested readings:

1. Monteiro MF, Lemos JG, Pontes FS, Silva AC, Silva MH, Silva NF, Souza LL, Uchôa DC, Pontes HA. Oral squamous cell carcinoma arising from areas of Graft-versus-host disease: A systematic review. Med Oral Patol Oral Cir Bucal. 2024 Jan 1;29(1):e135-e144.
2. Chaulagain CP, Sprague KA, Pilichowska M, Cowan J, Klein AK, Kaul E, Miller KB. Clinicopathologic characteristics of secondary squamous cell carcinoma of head and neck in survivors of allogeneic hematopoietic stem cell transplantation for hematologic malignancies. Bone Marrow Transplant. 2019 Apr;54(4):560-566.
3. Vale N, Pereira M, Mendes RA. Systemic Inflammatory Disorders, Immunosuppressive Treatment and Increase Risk of Head and Neck Cancers-A Narrative Review of Potential Physiopathological and Biological Mechanisms. Cells. 2023 Sep 1;12(17):2192.
4. Anak S, Yalman N, Bilgen H, Sepet E, Deviren A, Gürtekin B, Tunca F, Başaran B. Squamous cell carcinoma development in Fanconi anemia patients who underwent hematopoietic stem cell transplantation. Pediatr Transplant. 2020 Jun;24(4):e13706.
5. Hanna GJ, Kofman ER, Shazib MA, Woo SB, Reardon B, Treister NS, Haddad RI, Cutler CS, Antin JH, Van Allen EM, Uppaluri R, Soiffer RJ. Integrated genomic characterization of oral carcinomas in post-hematopoietic stem cell transplantation survivors. Oral Oncol. 2018 Jun;81:1-9.
